# Technology Implementation in Persons With Dementia: A Systematic Review and Meta-Analysis

**DOI:** 10.1093/geroni/igaa057.1012

**Published:** 2020-12-16

**Authors:** Sarah Hubner, Julie Blaskewicz Boron, Brenda Nguyen

**Affiliations:** University of Nebraska Omaha, Omaha, Nebraska, United States

## Abstract

Maintaining independence and quality of life (QOL) is a primary goal for adults. To support this goal, assistive and interactive technology (AIT) has been implemented to improve function and mitigate disease. To assess AIT effect on QOL in community-dwelling persons with dementia and mild cognitive impairment (MCI), a systematic review was performed, and articles were prepared for meta-analysis. Electronic database searches were carried out in PubMed, Cumulative Index for Nursing and Allied Health Literature, PsychINFO, and Web of Knowledge/Web of Science. Peer-reviewed journal articles published in English between January 2010 and February 2020 were included in the search. Studies investigated personal AIT use aimed at improving QOL (i.e. satisfaction/mood, functional ability, psychological/social function, independence). Technology was implemented in the home in everyday life. Studies were limited to those including community-dwelling participants aged 65+ with a diagnosis or report of MCI or dementia. Initial search resulted in 2624 total titles. After duplicate deletion, 1546 unique articles were identified. After title and abstract deletion, 60 articles were screened at full-text. After full-text screening, five usable articles remained. Usable studies presented: 1) a digital tablet companion, 2) a digital reminder calendar, 3) a medication-adherence bottle, 4) an automatic medication dispenser, and 5) a comprehensive tele-care computer system. These studies provide outcome measures focused on functional improvement and/or subjective QOL, informing future research in AIT implementation. Additionally, the severe paucity of applicable studies indicates a need for increased primary research on intersections between dementia and technology in the home environment.

